# Viral vectors based on bidirectional cell-specific mammalian promoters and transcriptional amplification strategy for use *in vitro *and *in vivo*

**DOI:** 10.1186/1472-6750-8-49

**Published:** 2008-05-16

**Authors:** Beihui Liu, Julian F Paton, Sergey Kasparov

**Affiliations:** 1 Department of Physiology and Pharmacology, Bristol Heart Institute, School of Medical Sciences, University of Bristol, Bristol, BS8 1TD, UK

## Abstract

**Background:**

Using cell-type-specific promoters to restrict gene expression to particular cells is an attractive approach for gene therapy, but often hampered by insufficient transcriptional activity of these promoters. Previous studies have shown that transcriptional amplification strategy (TAS) can be used to enhance the activity of such promoters without loss of cell type specificity. Originally TAS involved the use of two copies of a cell-specific promoter leading to generation of large expression cassettes, which can be hard to use given the space limitations of the conventional viral gene expression vectors.

**Results:**

We have now developed a new bidirectional lentiviral vector system, based on TAS that can enhance the transcriptional activity of human synapsin-1 (SYN) promoter and the compact glial fibrillary acidic protein (GfaABC_1_D) promoter. In the opposite orientation, a minimal core promoter (65 bp) derived from the human cytomegalovirus (CMV) was joined upstream of the SYN promoter or GfaABC_1_D promoter. This led to the formation of synthetic bidirectional promoters which were flanked with two gene expression cassettes. The 5' cassette transcribed the artificial transcriptional activator. The downstream cassette drove the synthesis of the gene of interest. Studies in both cell cultures and *in vivo *showed that the new bidirectional promoters greatly increased the expression level of the reporter gene. *In vivo *studies also showed that transgene expression was enhanced without loss of cell specificity of both SYN and GfaABC_1_D promoters.

**Conclusion:**

This work establishes a novel approach for creating compact TAS-amplified cell-specific promoters, a feature important for their use in viral backbones. This improved approach should prove useful for the development of powerful gene expression systems based on weak cell-specific promoters.

## Background

The widespread phenotype diversity within the central nervous system underscores the importance of restricting transgene expression to a specified target cell type [1-4]. Failure to do so results in gene expression in non target cells that confounds data interpretation and may lead to undesirable side effects. Restricting gene expression to a specified cell population within the brain by using cell-selective promoters remains an attractive approach [5,6]. In addition, cell-type-specific promoters are advantageous since they are less likely to activate host cell defense machinery and are less sensitive to cytokine-induced promoter inactivation than viral promoters [6]. As such, improved stability and longevity of gene expression can be expected.

**Figure 1:**
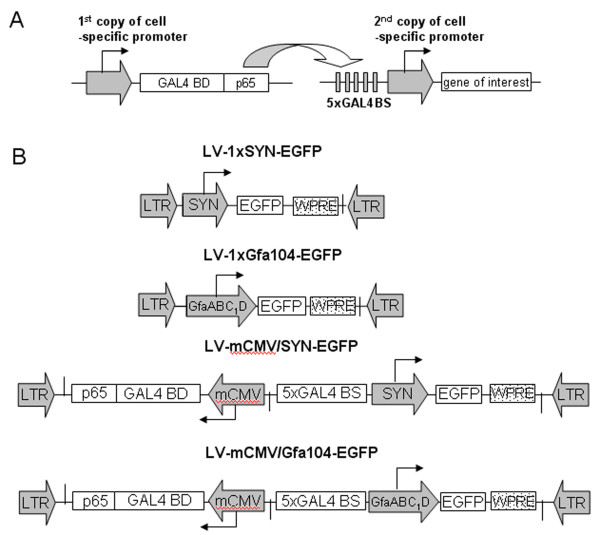
A: Schematic diagram of the TAS strategy. First copy of a cell-specific promoter was used to drive expression of a strong recombinant transactivator, for example GAL4BDp65 fusion protein which consisted of a part of the transcriptional activation domain of the NF-κB p65 protein fused to the DNA-binding domain of GAL4 protein from yeast. The GAL4BDp65 protein then interact with the unique GAL4 binding sequences upstream of the second copy of the cell-specific promoter leading to transactivation of the gene of interest and thus an enhancement of transcription. B: Layout of the lentiviral vectors used in this study. Abbreviations: LTR, lentiviral long terminal repeat; SYN, human synapsin 1 promoter (470 bp); GfaABC_1_D, a compact glial fibrillary acidic protein promoter (690 bp); mCMV, minimal CMV core promoter (65 bp); GAL4BDp65, a chimeric transactivator consisting of a part of the transactivation domain of the murine NF-κBp65 protein fused to the DNA binding domain of GAL4 protein from yeast; EGFP, enhanced green fluorescent protein; WPRE, woodchuck hepatitis post-transcriptional regulatory element.

The SYN and GfaABC_1_D promoter are two such cellular promoters that may offer cell specific gene expression in neurons and glia in the CNS, respectively. The SYN promoter has been extensively characterized and its 495-bp 5' flanking region has been shown to drive neuron-specific expression in various regions of the brain [7,8]. GfaABC_1_D promoter is a compact glial fibrillary acidic protein (GFAP) promoter with the size of 694-bp. It was derived from the conventional 2.2 kb human GFAP promoter [9]) by deleting 5' nucleotides -2163 to -1758 and an internal segment from -1255 to -133. GfaABC_1_D has expression properties in transgenic mice indistinguishable from the 2.2 kb version [10]. A general limitation of the applicability of cellular promoters, including the SYN and GfaABC_1_D promoters, has been their relatively weak transcriptional activity compared with viral promoters such as CMV promoter. TAS (also referred to as two step transcriptional amplification) has been proven to be an efficient strategy to enhance transgene expression from weak cell-specific promoters [5,11,12]. The basic principle of TAS is to use a cell-specific promoter to drive simultaneous expression of the desired transgene and a strong artificial transcriptional activator to potentiate transcription by binding to the specific binding sites introduced into the promoter (Figure 1A). Therefore, two copies of a cell-specific promoter were involved in this strategy, one to transcribe the transgene of interest and the other to express the transactivator. However, a limitation of such dual promoter system in the context of viral gene targeting is its size, which becomes an issue when longer promoters (e.g. > 2 kb) have to be used. Lentiviral vectors (LVV) and recombinant adenoviral vector (AVV) are two commonly used viral vectors in the CNS with packaging capacities of approximately 10 kb and 7 kb respectively [13,14]. Taking a recombinant adenovirus as an example, the maximum promoter sequence used in a dual promoter TAS system is around 2 kb leaving room for one medium-sized gene. On the other hand, it is well known that the size of the promoter sequence required for specific expression can be quite large, e.g., 5~6 kb and more [15,16]. Therefore, application of TAS in AVV and LVV is restricted to small promoters and short transgenes. To broaden the application of this strategy, it is highly desirable to reduce the overall size of the expression cassettes. This was the aim of the present study.

The recent demonstration of synthetic bidirectional promoters that mediate coordinate transcription of two mRNAs [17] prompted us to test whether this design is applicable to TAS. In synthetic bidirectional promoters a minimal core promoter is joined upstream to an efficient promoter positioned in the opposite orientation [17]. The rationale of the design was that upstream elements in the efficient promoter, when closely flanked by minimal promoters on both sides, drive transcriptional activity in both directions [18-22]. Earlier, Baron *et al*. (1995) constructed tetracycline-inducible bidirectional promoters by duplicating a minimal promoter on both sides of a series of Tet operator repeats to obtain exogenously regulated expression of two transgenes in a correlated, dose-dependent manner [23]. Here we applied bidirectional promoter design in combination with TAS *in vitro *and *in vivo*. We tested two cell-specific promoters, SYN and GfaABC_1_D promoters. The properties of these two promoters were described earlier.

## Results and discussion

Five self-inactivated HIV-derived lentiviral vectors (Figure 1B) were constructed for this study containing: (1) the EGFP reporter gene under the control of the SYN promoter alone (LV-1 × SYN-EGFP), (2) the EGFP reporter gene under the control of the GfaABC_1_D promoter alone (LV-1 × GfaABC_1_D-EGFP), (3) SYN-based bidirectional promoter driving the synthesis of the transcriptional activator GAL4p65 (for details about GAL4p65, refer to [12]) and the reporter gene EGFP (LV-mCMV/SYN-EGFP), (4) GfaABC_1_D-based bidirectional promoter driving the synthesis of the transcriptional activator GAL4p65 and the reporter gene EGFP (LV-mCMV/GfaABC_1_D-EGFP). LV-1 × SYN-EGFP and LV-1 × GfaABC_1_D-EGFP served as controls lacking the transcriptional activator GAL4p65. In LV-mCMV/SYN-EGFP and LV-mCMV/GfaABC_1_D-EGFP, a minimal CMV core promoter (mCMV, 65 bp) derived from pTRE-Tight-DsRed2 (Clontech) was joined in the opposite orientation to either the SYN or GfaABC_1_D promoter to form bidirectional promoters mCMV/SYN and mCMV/GfaABC_1_D. Two gene expression cassettes flanked the bidirectional promoters. The 5' cassette transcribed the strong GAL4p65 transactivator. The 3' cassette drove the synthesis of the reporter gene with 5 tandem GAL4 binding sequences at the 5' end of the specific promoter. Woodchuck hepatitis virus post-transcriptional regulatory element (WPRE [24-26]) was included in all of the four constructs to further enhance the expression level of the reporter gene. If the bidirectional promoters mCMV/SYN and mCMV/GfaABC_1_D are active in both directions, upstream product GAL4p65 would bind to GAL4 binding sequences introduced 5' of SYN or GfaABC_1_D promoter. This we anticipated would then lead to boosted expression of EGFP.

**Figure 2:**
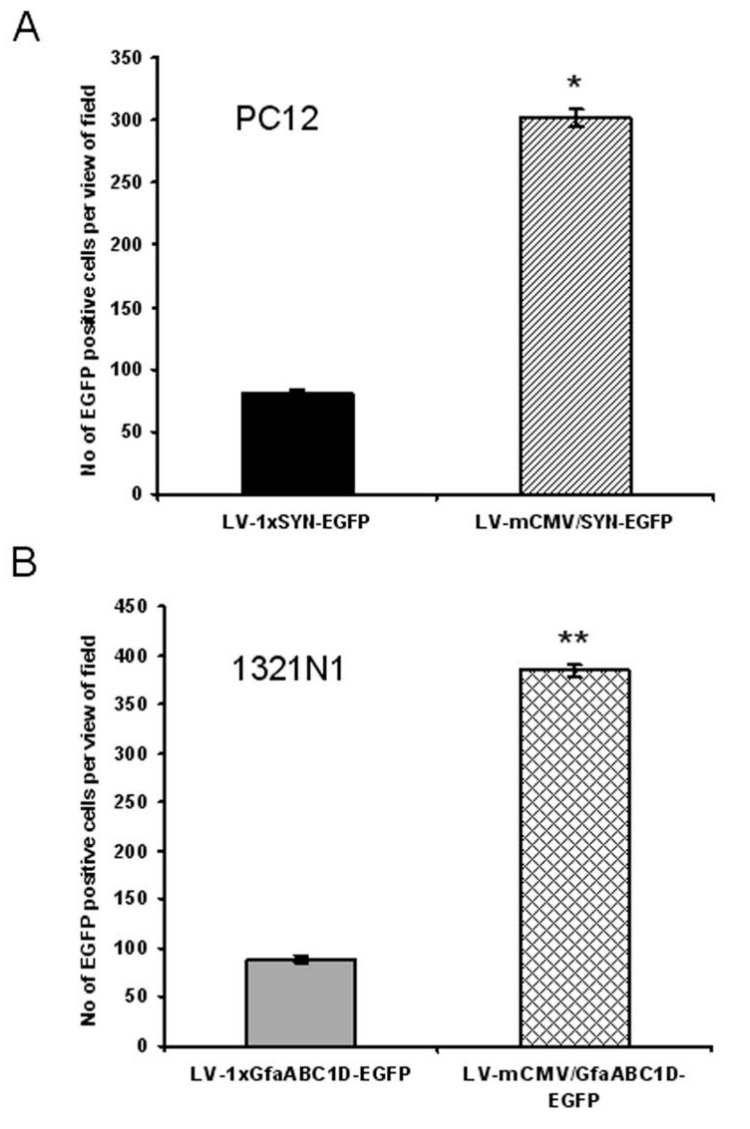
GAL4p65 augments EGFP expression from synthetic bidirectional SYN (A) and GfaABC_1_D (B) promoters in cell lines. Number of EGFP positive cells was counted per field under the magnification of 100. For each virus, three wells were transduced and six fields were selected randomly for cell counting. MOI for each virus was 5. * p < 0.01, compared with LV-SYN-EGFP; ** p < 0.001, compared with LV-1 × GfaABC_1_D-EGFP. An unpaired *t *test was applied for comparisons between two groups. The differences were considered significant at P < 0.05. All values in the figures refer to mean ± SD.

We initially analyzed the performance of the bidirectional constructs in cell culture. Neuron-derived PC12 cells were transduced with LV-1 × SYN-EGFP and LV-mCMV/SYN-EGFP while glia-derived 1321N1 cells were transduced with LV-1 × GfaABC_1_D-EGFP and LV-mCMV/GfaABC_1_D-EGFP at MOI of 5. Bidirectional constructs produced significantly more EGFP-positive cells in both PC12 and 1321N1 cells. Thus the number of EGFP-positive PC12 cells from LV-mCMV/SYN-EGFP was increased ~3.7-fold (Figure 2A) as compared to that from LV-1 × SYN-EGFP. Similarly, expression from LV-mCMV/GfaABC_1_D-EGFP was increased ~4.3-fold in 132 1N1 cells (Figure 2B) when compared with that from LV-1 × GfaABC_1_D-EGFP. These results confirmed the boosted gene expression of both bidirectional TAS-based LVV systems.

**Figure 3:**
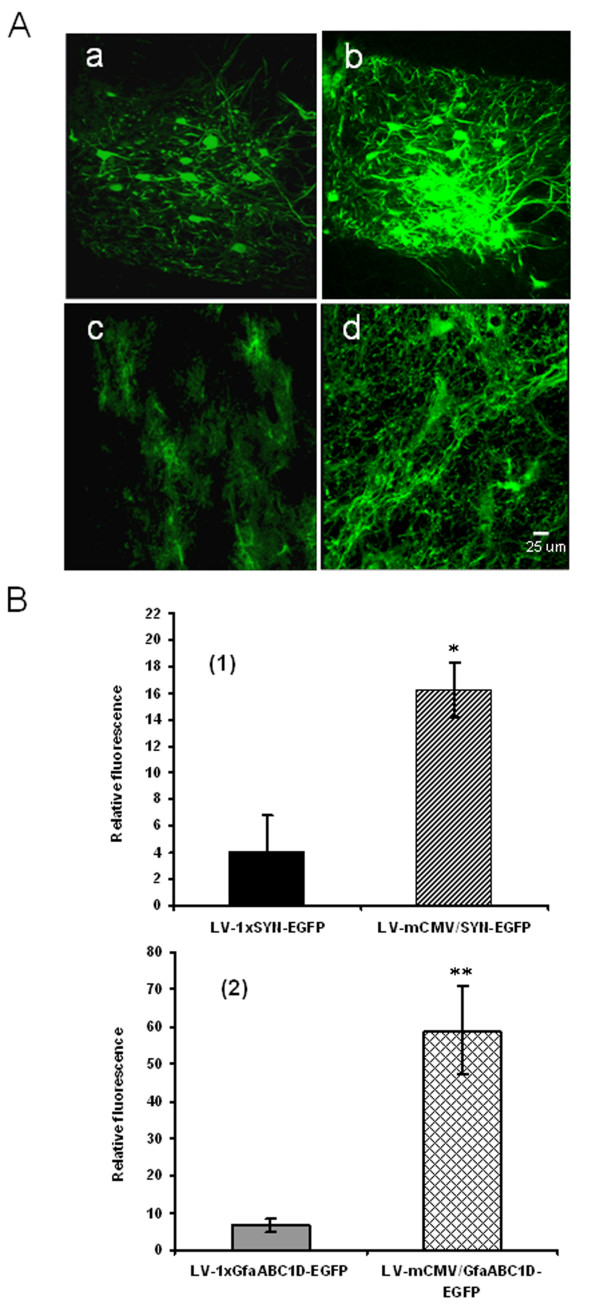
GAL4p65 augments EGFP expression from synthetic biodirectional SYN and GfaABC_1_D promoters in the rat brain *in vivo*. A: Representative images from rats injected with LV-1 × SYN-EGFP (a), LV-mCMV/SYN-EGFP (b), LV-1 × GfaABC_1_D-EGFP (c) and LV-mCMV/GfaABC_1_D-EGFP (d). B: Assessing EGFP transgene expression level *in vivo*. (1): Relative EGFP fluorescence levels in rats transduced with LV-1 × SYN-EGFP or LV-mCMV/SYN-EGFP (n = 3). NIH ImageJ was used to quantitatively compare the relative EGFP fluorescence levels. Four sections surrounding the injection tract per rat were selected randomly and three fields from each section were used. (2): Relative EGFP fluorescence levels in rats transduced with LV-1 × GfaABC_1_D-EGFP or LV-mCMV/GfaABC_1_D-EGFP (n = 3). * p < 0.01, compared with LV-SYN-EGFP; ** p < 0.001, compared with LV-1 × GfaABC_1_D-EGFP. An unpaired *t *test was applied for comparisons between two groups. The differences were considered significant at p < 0.05. All values in the figures refer to mean ± SD.

We next evaluated the performance of the new vectors *in vivo *in the rat brain. LVV were stereotaxically injected into the hypoglossal motor nucleus. To allow for direct comparison, we set the dose for each virus for one rat as 10^6 ^infectious units and transgene expression was scored one week postinjection. As shown in Figure 3A, significantly stronger EGFP expression was observed from LV-mCMV/SYN-EGFP and LV-mCMV/GfaABC_1_D-EGFP than that from LV-1 × SYN-EGFP and LV-1 × GfaABC_1_D-EGFP. NIH ImageJ was used to quantitatively compare the relative EGFP fluorescence levels. We observed a ~4-fold increase in the level of fluorescence in tissues transduced by LV-mCMV/SYN-EGFP than by LV-1 × SYN-EGFP [Figure 3B(1)] and ~9-fold increase by LV-mCMV/GfaABC_1_D-EGFP than by LV-1 × GfaABC_1_D-EGFP [Figure 3B(2)].

**Figure 4:**
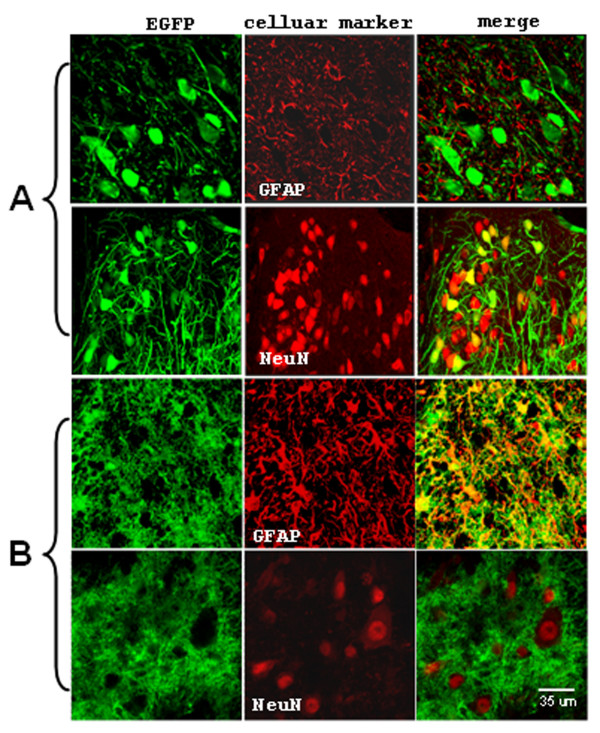
Specificity of the transcriptional amplification strategy based on synthetic bidirectional SYN (A) and GfaABC_1_D (B) promoters as demonstrated by immunostaining for neuronal antigen NeuN and glial antigen GFAP. LV-mCMV/SYN-EGFP (A) and LV-mCMV/GfaABC_1_D-EGFP (B) were stereotaxically injected into the rat hypoglossal motor nucleus at the dose of 1 × 10^6 ^IU viruses (n = 3). Tissues were collected 7 days after lentivirus injection. Frozen coronal transverse sections were used for NeuN and GFAP immunostaining.

To determine whether the cell-type specificity was preserved in bidirectional promoters, we performed immunohistochemical staining with antibodies against the neuron-specific nuclear protein (NeuN) to visualize neurons and antibodies against the glial fibrillary acidic protein (GFAP) to visualize astrocytes. Essentially, all EGFP-positive cells from rats injected with LV-mCMV/SYN-EGFP were NeuN-positive, whereas none of them were stained positively for GFAP, indicating exclusive neuron specific expression (Figure 4A). In contrast, for LV-mCMV/GfaABC_1_D-EGFP injected rats, EGFP-positive cells were positively stained with GFAP, while in no case was there co-localization of EGFP fluoresecence with NeuN. This confirmed cell-specific expression of EGFP that was restricted to glia (Figure 4B). Thus, we have demonstrated that bidirectional promoter design can be applied successfully to TAS to significantly boost the transcriptional activity of two weak cellular promoters without changing their cell-type specificity.

Although we used two heterogeneous core promoters other investigators reported that a unidirectional promoter may be bidirectionalized by fusing either a homogeneous or heterogenous minimal core promoter at its 5' end in the opposite orientation [17,27-29]. Apart from cell-specific promoters, which can be made bidirectional as demonstrated in the current study, constitutive and inducible promoters can also be bidirectionalized [17,23,28]. Thus, we believe that the ability to confer bidirectional expression to a promoter is not a special feature of just a few selected promoters. Future studies would benefit from applying the bidirectional TAS as described in this study to create potent phenotype specific-viral gene expression systems.

Few endogenous bidirectional promoters have been described until recently. Surprisingly, the human genome survey disclosed a prevalence of bidirectional gene pairs, representing more than 10% of the genes in the genome, whose transcription sites are separated by less than 1000 base pairs [30-32]. The significance of divergent gene organization is uncertain. Takai and Jones hypothesized that divergent gene organization might stem from the evolution of the human genome from a more compact genome [31]. Alternatively, divergently transcribed gene pairs and their bidirectional promoters may act as unique constructs to coordinate gene expression. Although the structural and functional implications of the widespread occurrence of bidirectional promoters in the human genome are not fully understood, transcription of these clusters of closely spaced genes may contribute to enhance communication and interplay between promoter elements and transcriptional factors [29]. Therefore, the synthetic bidirectional promoter design validated in this study may mimic a well-represented and evolutionarily conserved feature of eukaryotic transcription, providing a structural architecture for their robust performance.

## Conclusion

Our study presents an updated TAS with improved suitability for viral vector-based expression systems. This strategy should be useful for constructing powerful gene expression systems based on other weak cell-specific promoters of larger sizes. We have also constructed AVV based on similar expression cassettes and confirmed their improved performance, although these results are not presented in this communication. The LVV based on TAS and the bidirectional promoters as constructed in this study will be of value for the exploration of *in vivo *gene function and future gene therapy applications.

## Methods

### Plasmid construction

Four lentiviral plasmids (Table 1) were constructed based on the improved lentiviral shuttle vector pTYF-SW-Linker backbone [33]. To construct the LV-1 × SYN-EGFP shuttle vector pTYF-1 × SYN-EGFP, we first inserted the *Not*I/*Cla*I PCR fragment of WPRE amplified from woodchuck hepatitus B virus genomic DNA (NCBI access no: J04514) into the pTYF-SW-linker. An EGFP PCR fragment, amplified from pEGFP-C1 (Clontech, Palo Alto, CA, USA) was then cloned into the *Spe*I/*Not*I sites. Finally, the 495-bp human SYN promoter PCR product from pSYN1 (kindly provided by Dr.S.Kűgler, University of Gőttingen, Germany) was inserted between *Mlu*I/*Spe*I sites. The LV-1 × GfaABC_1_D-EGFP shuttle vector pTYF-1 × GfaABC_1_D-EGFP was obtained by replacing the SYN promoter in pTYF-1 × SYN-EGFP with the GfaABC_1_D PCR product from pGfaABC_1_D-LacZ (kindly provided by Prof. M Brenner, Department of Neurobiology, University of Alabama at Birmingham, USA, for details please refer to [10]) between *Mlu*I and *Spe*I sites. Three cloning steps were necessary to generate pTYF-mCMV/SYN-EGFP and pTYF-mCMV/GfaABC_1_D-EGFP. First, a PCR product containing the minimal CMV promoter, GAL4p65 and SV40pA was amplified from pBD-NF-κB (a control plasmid from the mammalian two-hybrid assay kit, Stratagene) and inserted between *Mlu*I/*Spe*I sites. WPRE PCR product was then cloned between *Not*I/*Cla*I. Finally, the *Nhe*I/blunt/*Not*I fragment from pTYF-1 × SYN-EGFP or pTYF-1 × GfaABC_1_D-EGFP was inserted into the resultant plasmid from the above two steps previously treated with *Mlu*I/blunt/*Not*I to produce pTYF-mCMV/SYN-EGFP and pTYF-mCMV/GfaABC_1_D-EGFP respectively.

**Table 1:** Lentiviral shuttle vectors used in the current study.

Name	Promoter	Transgene product
pTYF-1 × SYN-EGFP	SYN with GAL4 binding sites	EGFP
pTYF-mCMV/SYN-EGFP	bidirectional promoter mCMV/SYN	GAL4p65 and EGFP
pTYF-1 × GfaABC_1_D-EGFP	GfaABC_1_D with GAL4 binding sites	EGFP
pTYF-mCMV/GfaABC_1_D-EGFP	bidirectional promoter mCMV/GfaABC_1_D	GAL4p65 and EGFP

### Production of lentiviral vectors

The LVV system used in this study is derived from HIV-1 and pseudotyped with the vesicular stomatitis virus coat glycoprotein. Stocks were produced by transient cotransfection of the shuttle plasmids, the packaging vector pNHP, and the envelope plasmid pHEF-VSVG in HEK293FT cells (Invitrogen, Carlsbad, CA, USA). Viral concentration and titration were carried out as described earlier [34].

### Cell culture and in vitro lentiviral vector transduction

The *in vitro *transduction experiments were carried out in neurone-derived rat pheochromocytoma PC12 cell line (ATCC, No. CRL-1721) and 1321N1 glial cell line from human brain astrocytoma (ECACC, No. 86030402). PC12 cells were grown in Dulbecco's modified Eagle's medium (DMEM) supplemented with 10% heat-inactivated FBS and 5% horse serum. 1321N1 cells were cultured in DMEM medium supplemented with 10% heat-inactivated FBS. Cells were split one day prior to transfection and plated in 24-well plates at a cell density of 5 × 10^4 ^per well. After overnight incubation, cells were transduced with lentiviral vectors in the presence of polybrene (8 ug/ml). Cells were then washed in PBS and were cultured in DMEM for a further 48 hrs.

### In vivo lentiviral vector transduction into the rat hypoglossal motor nucleus

Lentiviral vectors were stereotaxically injected into the hypoglossal motor nucleus of male Wistar rats (250–300 g). All procedures were carried out according to the Home Office animals Scientific Procedures Act 1986, UK. Animals were deeply anaesthetized with an intramuscular injection of ketamine (60 mg/kg) and medetomidine (250 μg/kg). They were placed in a stereotaxic head holder and the caudal dorsal medulla was exposed through a midline incision in the dorsal neck. A total of six microinjections of viral vector were made bilaterally at the level of the calamus scriptorius and 400 μm rostral and caudal to it, 300–500 μm from the midline and 450–550 μm ventral to the dorsal surface of the medulla as described previously [33]. The injection rate was 0.5 μl/min and the needle was allowed to remain *in situ *for 5 min before being slowly retracted at the end of each injection. To allow for direct comparison, we set the dose for each virus for one rat as 10^6 ^infectious units. At 7 days after injection, rat brain stems were collected. Three rats were used for each virus. Frozen coronal sections of each brain were cut at 40 um thickness and used for imaging.

### Immunohistochemistry analysis

Frozen coronal transverse sections were cut at 40 μm thickness and free-floating sections were washed 3 times for 20 min in 0.1 M PBS at pH 7.4 containing 0.2% Triton X-100, then blocked with 5% normal horse serum (NHS) in PBS for 1 h. Sections were then incubated overnight with monoclonal antibody against NeuN or GFAP (both from Chemicon International, USA; dilution 1:500). This was followed by 2 hrs incubations in biotinylated donkey-anti mouse F(ab)_2 _fragments (1: 500, Jackson Immunolabs, PA, USA) and 2% NHS in PBS, then ExtrAvidin-Cy3 in PBS (1: 1000, Sigma). They were collected on gelatincoated slides with non-quenching mounting medium Vectashield (Vector labs, CA, USA). Images were captured using an Inverted Leica Confocal Imaging Spectrophotometer System (TCS-SP2) at 1–2 μm intervals through the thickness of the section. The two channels (EGFP and Cy3) were scanned separately to avoid "bleed" of fluorescence between channels and merged using Leica software.

## Authors' contributions

BHL was responsible for experimental design and completion of all laboratory work presented in this article. SK contributed to the conception of the study and participated in all stages of the work. JFRP helped to plan and coordinate the study and helped draft the manuscript. All authors have read and approved the final manuscript.
